# Hydrogen-Rich Water Potentiates Cannabinoid- and Gabapentinoid-Induced Analgesia in Neuropathic Pain

**DOI:** 10.3390/ijms262412155

**Published:** 2025-12-18

**Authors:** Nuria Andrea Tort, Sylmara Esther Negrini-Ferrari, Olga Pol

**Affiliations:** 1 Grup de Neurofarmacologia Molecular, Institut de Recerca Sant Pau (IR SANT PAU), Sant Quintí 77-79, 08041 Barcelona, Spain; 2 Grup de Neurofarmacologia Molecular, Institut de Neurociències, Universitat Autònoma de Barcelona, 08193 Barcelona, Spain

**Keywords:** neuropathic pain, molecular hydrogen, cannabinoids, gabapentinoids, analgesia

## Abstract

Neuropathic pain (NP) is a complex and disabling condition that often requires long-term treatment with high doses of pharmacological agents, frequently resulting in significant adverse side effects. Therefore, safer and more effective therapeutic approaches are urgently needed. Molecular hydrogen, recognized for its antioxidant and anti-inflammatory actions, may act as a valuable adjunct to conventional analgesics. This study examined whether hydrogen-rich water (HRW) could potentiate the analgesic effects of JWH-133, a selective cannabinoid receptor type 2 agonist, and pregabalin, a gabapentinoid, in male C57BL/6 mice with NP induced by chronic constriction of the sciatic nerve. Mechanical allodynia, thermal hyperalgesia, and cold allodynia were assessed following separate or combined administration of HRW with JWH-133 or pregabalin. Western blot analyses of dorsal root ganglia measured markers of oxidative stress (4-HNE), inflammation (NLRP3), synaptic plasticity (p-ERK), and nociceptive signaling (p-AKT). Each treatment reduced pain-like behaviors in a dose-dependent manner, while co-administration of HRW with JWH-133 or pregabalin produced greater analgesic effects. Combined treatments also diminished oxidative stress, inflammation, maladaptive neural changes and nociceptive pathways activated by peripheral nerve injury. These findings suggest HRW as a promising adjuvant to cannabinoid and gabapentinoid therapies, potentially improving efficacy and reducing high-dose drug-related adverse effects.

## 1. Introduction

Neuropathic pain (NP) is a complex, often chronic condition caused by a lesion or disease of the somatosensory system. It originates from damage to nerves involved in the neural pathways that transmit sensory information from the body to the brain [1,2].

NP also leads to peripheral and central sensitization, resulting in an increased response to painful stimuli (hyperalgesia) and pain in response to normally non-painful stimuli (allodynia). These alterations make NP not only a daily and persistent struggle for patients who suffer from it but also a significant economic burden on healthcare systems [1,3]. Moreover, the current pharmacological treatments are often ineffective, requiring high doses for prolonged periods of time, which lead to numerous side effects [4,5].

One of the main mechanisms underlying NP caused by peripheral nerve injury is thought to be the generation of ectopic impulses in nociceptive primary sensory neurons (nociceptors), either at the site of the injury or in the dorsal root ganglia (DRG) [1]. These abnormal impulses transmitted to the central nervous system are interpreted by the brain as pain signals, leading to NP. A wide variety of molecular mechanisms are involved in the development of NP, one of them is inflammation. The NOD-like receptor family pyrin domain-containing 3 (NLRP3) inflammasome, a multiprotein complex that triggers the release of pro-inflammatory cytokines such as IL-1β, is the main intracellular sensor of inflammation. Upregulation of NLRP3 expression has been observed in the DRG of animals with NP, suggesting that peripheral activation of this inflammasome plays a key role in the contribution of inflammation to the pathogenesis of NP [6,7].

Oxidative stress also plays a crucial role in the development of NP. Damaged peripheral sensory neurons undergo mitochondrial dysfunction, leading to the overproduction of reactive oxygen species (ROS), which contribute to NP through several molecular mechanisms, such as facilitating the activation of macrophages in peripheral nerves that potentiate the inflammatory responses. In addition, they also cause direct neuronal damage, enhance central sensitization, and modulate nociceptive signaling, altogether contributing to the progress and maintenance of NP [8]. As a consequence, increased levels of oxidative markers, such as 4-HNE, were detected in the DRG and some brain areas of sciatic nerve-injured mice [7].

Both inflammation and oxidative stress contribute to synaptic plasticity changes in nociceptors and glial cells, leading to their central and peripheral sensitization, resulting in hyperalgesia and allodynia. In persistent pain conditions, there is an increase in the MAPK signaling pathways in glial cells, including the ERK 1/2 (extracellular signal-regulated kinase 1 and 2), p38, and c-Jun N-terminal kinase pathways. These changes promote neuroinflammation and, consequently, pain hypersensitivity. For example, following nerve injury, ERK 1/2 has been shown to be initially phosphorylated in microglia, suggesting its implication in the induction of chronic pain, and later in astrocytes, where it is thought to be involved in the maintenance of this state [8,9].

Phosphoinositide 3-kinase/protein kinase B (PI3K/AKT) signaling pathway has been reported to participate in the onset of hypersensitivity to pain, particularly by contributing to the spinal cord sensitization, a crucial region modulating the transmission of nociceptive signals to the central nervous system [10]. Experimental evidence demonstrated that pharmacological inhibition of either PI3K or Akt attenuates NP behaviors, including mechanical allodynia and thermal hyperalgesia, in animal models of peripheral nerve injury [11]. Elevated levels of p-Akt have been observed in both the DRG and the spinal cord following nerve ligation, further supporting the contribution of this pathway to central and peripheral sensitization [12].

First-line pharmacological management of NP comprised the use of gabapentinoids (gabapentin and pregabalin) and antidepressants such as tricyclic antidepressants and serotonin–norepinephrine reuptake inhibitors [1]. When these are insufficient or poorly tolerated, topical or systemic local anesthetics, e.g., lidocaine patches and high-concentration capsaicin patches, are prescribed. Finally, third-line treatments, including opioids or cannabinoids, may be considered, although these options are often limited by concerns about safety, tolerability, and long-term benefit [13,14].

Despite their prominent role as first-line treatments, gabapentinoids are not universally effective across all NP subtypes and are frequently associated with side effects such as dizziness, somnolence, and impaired attention or working memory, which can significantly impact patient adherence and daily functioning [3,14]. These limitations highlight the need to refine gabapentinoid-based approaches through optimized dosing strategies, personalized treatment selection, or combination therapies designed to enhance efficacy while minimizing adverse outcomes.

Cannabinoid receptor agonists have shown therapeutic efficacy in the treatment of NP. Preclinical investigations have demonstrated that selective activation of cannabinoid type-2 receptors (CB2R) elicits antinociceptive effects in multiple models of NP [15,16]. Nonetheless, their clinical effectiveness is limited, and their application is frequently associated with considerable adverse effects, including diminished attention and working memory [3,17]. Therefore, additional research into novel therapeutic strategies that incorporate combined treatments to augment analgesic efficacy while reducing side effects is essential.

Recent studies have highlighted the therapeutic potential of molecular hydrogen (H_2_) in the treatment of NP and other neurological disorders [18]. Experimental evidence indicates that H_2_ administration can markedly reduce mechanical allodynia and thermal hyperalgesia, symptoms of NP [19]. The analgesic and neuroprotective effects of H_2_ are primarily attributed to its antioxidant, anti-apoptotic, and anti-inflammatory properties, which collectively attenuate oxidative stress and neuroinflammatory signaling associated with chronic pain states [20,21]. H_2_ can be efficiently administered via injection of hydrogen-rich water (HRW), facilitating accurate dosing and enhanced H_2_ delivery while exhibiting minimal side effects or tolerance concerns [22]. Therefore, HRW represents a promising medicinal approach, but further research to explore the synergistic effects of H_2_ with conventional NP therapies is required.

The treatment of NP remains extremely challenging. Despite decades of research, 30–50% of patients are resistant to commonly used therapeutic strategies. The chronic nature of the condition and the high doses required for symptom control frequently result in undesirable side effects [14]. That is why combined therapies that manage to reduce the high doses required by current treatments are receiving more interest. These approaches aim to enhance the analgesic efficacy while minimizing adverse effects, thereby improving patients’ quality of life and adherence to therapy.

In this study, we explore the combination of HRW with either JWH-133, a selective CB2R agonist, or pregabalin, a gabapentinoid, as new alternatives for the management of NP. Therefore using a mice model of NP induced by the chronic constriction injury (CCI) of the sciatic nerve, we evaluated: (i) the dose-responses effects produced by the intraperitoneal administration of different doses of HRW, JWH-133 and pregabalin in the mechanical allodynia, thermal hyperalgesia and cold allodynia provoked by CCI, (ii) the effectiveness of the combination of a low dose of HRW with either JWH-133 or pregabalin on inhibiting the nociceptive responses caused by CCI and (iii) the molecular pathways involved in the actions of these treatments, alone and combined, through assessing the expression of proteins related to oxidative stress, inflammation, plasticity changes and nociceptive signaling pathways by using Western blot analysis.

## 2. Results

### 2.1. Validation of the CCI Mouse Model

At 28 days post-surgery, different tests were performed to confirm the development of the sensory manifestations associated with NP. CCI mice showed mechanical allodynia, thermal hyperalgesia and cold allodynia (Table 1). Thus, a significant reduced threshold of the ipsilateral hind paw withdrawal to von Frey filament stimulation, a reduced withdrawal threshold of the ipsilateral hind paw in response to a thermal stimulus, and an enhanced number of ipsilateral hind paw lifts caused by cold stimulus were observed in CCI mice as compared to their respective SHAM-operated mice (*p* < 0.05, unpaired Student’s *t*-test). These results validate the successful induction of the CCI model.

### 2.2. Dose-Dependent Antinociceptive Effects of HRW in Animals with NP

**Figure 1:**
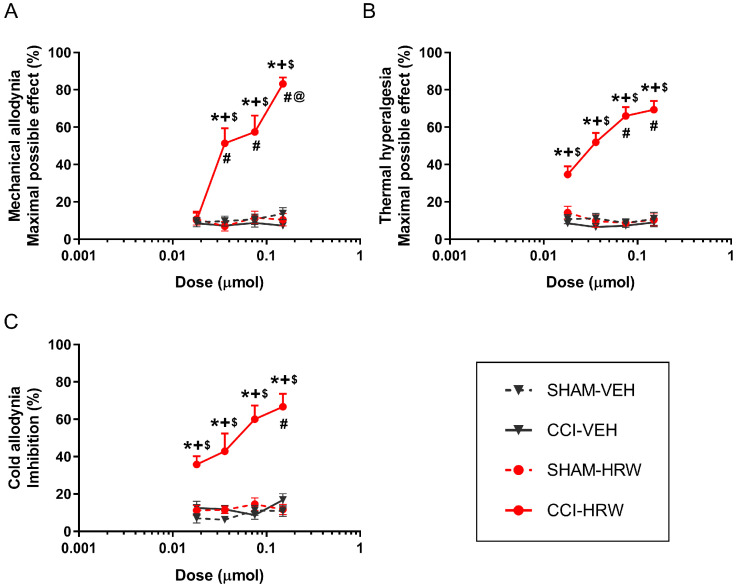
Dose-dependent antinociceptive effects of HRW or VEH in the ipsilateral paws of CCI- and SHAM-operated mice. The effects of different doses (0.018, 0.036, 0.075, and 0.150 μmol) of HRW or VEH, given intraperitoneally (logarithmic axis), are represented in the *y*-axis as maximal possible effect (%) for (**A**) mechanical allodynia and (**B**) thermal hyperalgesia and as inhibition (%) for (**C**) cold allodynia. The effects of HRW or VEH in the ipsilateral paws of SHAM-operated animals and those of VEH in the ipsilateral paws of CCI mice were also represented. In each test, each symbol denotes significant differences versus * SHAM-HRW, + SHAM-VEH, $ CCI-VEH, # CCI-HRW-0.018 μmol, and @ CCI-HRW-0.036 μmol (*p* < 0.05; one-way ANOVA followed by Student-Newman-Keuls post hoc test). The results are presented as mean values ± SEM; n = 6 animals per dose/group.

Our results indicated that the intraperitoneal administration of increasing doses of HRW (0.018, 0.036, 0.075, and 0.150 μmol) dose-dependently inhibited the mechanical allodynia, thermal hyperalgesia and cold allodynia caused by CCI (Figure 1).

The antiallodynic and antihyperalgesic effects generated by 0.036, 0.075, and 0.150 μmol of HRW in CCI mice were higher than those generated by this treatment in SHAM-operated animals or by VEH in CCI- and SHAM-operated mice (*p* < 0.0001; one-way ANOVA; Figure 1A–C). In the same line, the mechanical antiallodynic effects produced by these doses were higher than those produced by 0.018 μmol of HRW in CCI mice (Figure 1A), and the effects of 0.150 μmol of HRW were greater compared to those produced by 0.036 μmol in CCI mice. Regarding thermal hyperalgesia and cold allodynia, the inhibitory effects produced by high doses of HRW, such as 0.150 and/or 0.075 μmol, were also greater than those made by this treatment at 0.018 μmol in CCI-mice (*p* < 0.0001; one-way ANOVA; Figure 1B,C). In all tests, the maximal inhibitory effect was produced by a 0.150 μmol dose of HRW.

### 2.3. Dose-Dependent Antinociceptive Effects of JWH-133 in Animals with NP

**Figure 2:**
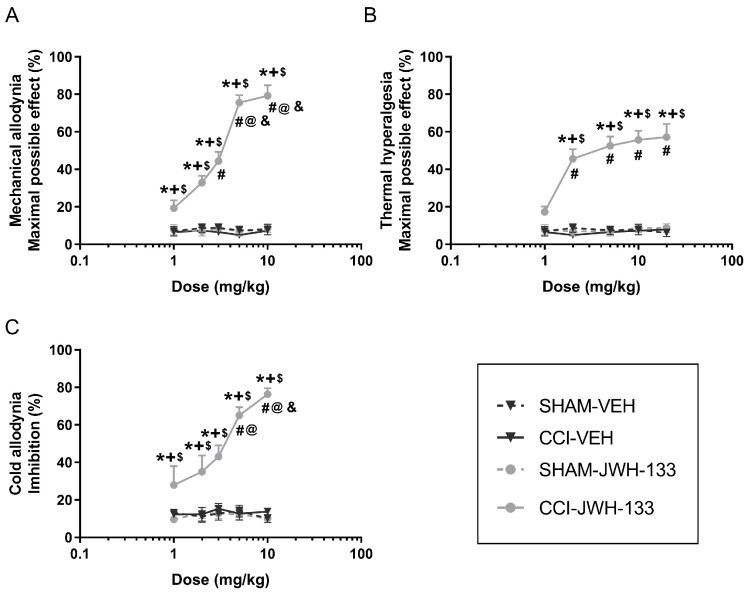
Dose-dependent antinociceptive effects of JWH-133 or VEH in the ipsilateral paws of CCI- and SHAM-operated mice. The effects of different doses (1, 2, 3, 5, 10, and 20 mg/kg) of JWH-133 or VEH, given intraperitoneally (logarithmic axis), are represented in the *y*-axis as maximal possible effect (%) for (**A**) mechanical allodynia and (**B**) thermal hyperalgesia and as inhibition (%) for (**C**) cold allodynia. The effects of JWH-133 or VEH in the ipsilateral paws of SHAM-operated animals and those of VEH in the ipsilateral paws of CCI mice were also represented. In each test, each symbol denotes significant differences versus * SHAM-JWH-133, + SHAM-VEH, $ CCI-VEH, # CCI-JWH-133 1 mg/kg, @ CCI-JWH-133 2 mg/kg, and & CCI-JWH-133 3 mg/kg (*p* < 0.05; one-way ANOVA followed by Student-Newman-Keuls post hoc test). The results are presented as mean values ± SEM; n = 6 animals per dose/group.

JWH-133 was also administered intraperitoneally at doses of 1, 2, 3, 5, 10, and/or 20 mg/kg, and results showed an inhibitory effect on the mechanical allodynia, thermal hyperalgesia and cold allodynia in a dose-dependent manner (Figure 2A–C).

Values in the CCI-JWH-133 mice group were significantly different from those in control groups (SHAM-VEH, CCI-VEH and SHAM-JWH-133) in all the pain-related responses tested (*p* < 0.0001; one-way ANOVA; Figure 2A–C), except for the 1 mg/kg dose in the thermal hyperalgesia test, which did not show any significant difference compared to its respective control groups (Figure 2B). In the mechanical and cold allodynia, the inhibitory actions of JWH-133 at 10 and/or 5 mg/kg were significantly higher than those produced by 1, 2, and/or 3 mg/kg doses (*p* < 0.0001; one-way ANOVA; Figure 2A,C) while the mechanical antiallodynic effects of this drug at 3 mg/kg were only different from those of it at 1 mg/kg in CCI mice (Figure 2A). In the thermal hyperalgesia, no significant differences were found between the effects produced by 2, 5, 10, and 20 mg/kg of JWH-133 in CCI mice, although all of them were significantly different from the effects produced by 1 mg/kg (*p* < 0.0001; one-way ANOVA; Figure 2B). The maximal effect of JWH-133 was observed at 10 mg/kg for allodynia and 20 mg/kg for hyperalgesia.

### 2.4. Dose-Dependent Antinociceptive Effects of Pregabalin in Animals with NP

**Figure 3:**
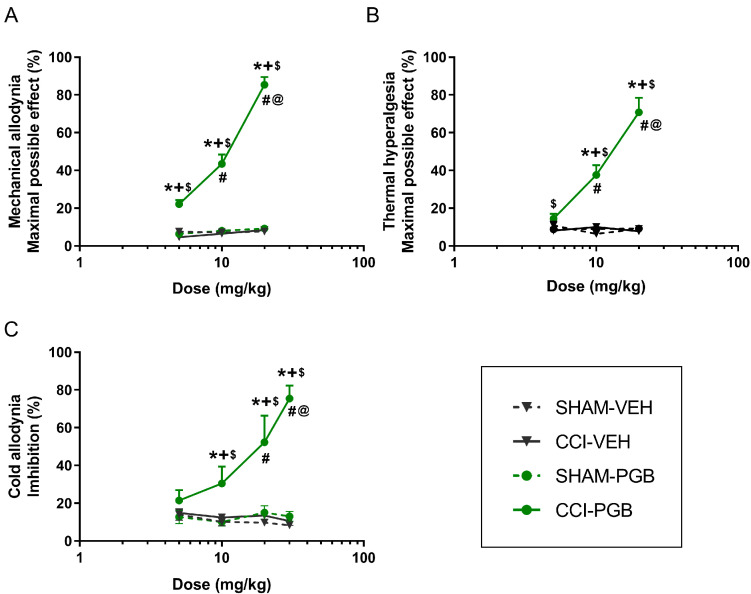
Dose-dependent antinociceptive effects of pregabalin or VEH in the ipsilateral paws of CCI- and SHAM-operated mice. The effects of different doses (5, 10, 20, and/or 30 mg/kg) of pregabalin (PGB) or VEH, given intraperitoneally (logarithmic axis), are represented in the *y*-axis as maximal possible effect (%) for (**A**) mechanical allodynia and (**B**) thermal hyperalgesia and as inhibition (%) for (**C**) cold allodynia. The effects of pregabalin or VEH in the ipsilateral paws of SHAM-operated animals and those of VEH in the ipsilateral paws of CCI mice were also represented. In each test, each symbol denotes significant differences versus * SHAM-PGB, + SHAM-VEH, $ CCI-VEH, # CCI-PGB 5 mg/kg, @ CCI-PGB 10 mg/kg (*p* < 0.05; one-way ANOVA followed by Student-Newman-Keuls post hoc test). The results are presented as mean values ± SEM; n = 6 animals per dose/group.

Injection of pregabalin at doses of 5, 10, 20, and/or 30 mg/kg in CCI mice also resulted in a dose-dependent inhibitory effect on the mechanical allodynia, thermal hyperalgesia and cold allodynia (Figure 3A–C).

In mechanical allodynia, all pregabalin doses tested in CCI mice were significantly different from those in control groups (SHAM-VEH, CCI-VEH and SHAM-PGB) (*p* < 0.0001; one-way ANOVA; Figure 3A). In thermal hyperalgesia, the inhibitory effects produced by 10 and 20 mg/kg of pregabalin also differed significantly from all control groups (*p* < 0.0001; one-way ANOVA; Figure 3B). Moreover, the antihyperalgesic effects of 20 mg/kg of pregabalin were higher than those produced by 5 and 10 mg/kg of this drug, while those of 10 mg/kg were only different from those produced by 5 mg/kg. In cold allodynia, 10, 20, and 30 mg/kg of pregabalin showed significant differences compared to any of the control groups, while the inhibitory effects of 30 mg/kg were significantly different from those produced by lower doses of this drug (5 and 10 mg/kg) (*p* < 0.0001; one-way ANOVA; Figure 3C) and of 20 mg/kg from those of 5 mg/kg of this anticonvulsant. Finally, the inhibitory actions of 20 mg/kg (mechanical allodynia and thermal hyperalgesia; Figure 3A,B) and 30 mg/kg of pregabalin (cold allodynia; Figure 3C) were greater than those made by 10 mg/kg of this drug (*p* < 0.0001; one-way ANOVA).

The maximal inhibitory effects of pregabalin in mice with NP were observed at 20 mg/kg for the mechanical allodynia and thermal hyperalgesia (Figure 3A,B) and at 30 mg/kg for the cold allodynia (Figure 3C).

In all the dose–response analysis experiments, CCI and SHAM mice treated with VEH, as well as SHAM mice treated with either HRW, JWH-133, or pregabalin, did not show significant changes between them (Figure 1A–C, Figure 2A–C and Figure 3A–C).

### 2.5. The Antinociceptive Effects of the Co-Treatment of HRW and JWH-133 in Animals with NP

**Figure 4:**
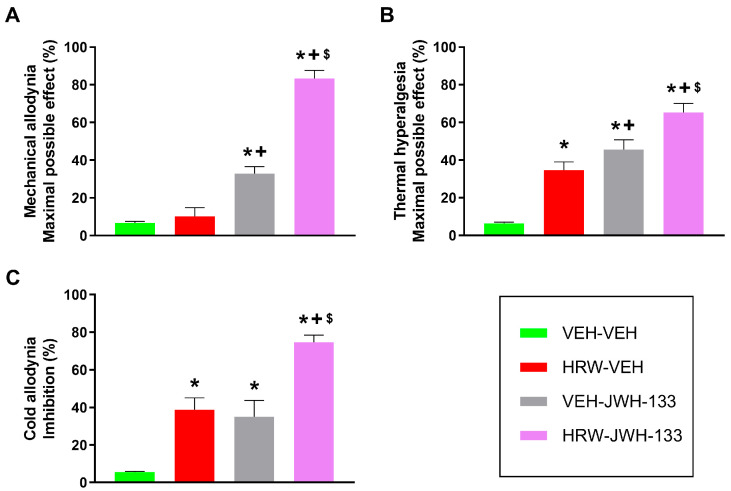
The analgesic actions of the combined treatment of HRW and JWH-133 in animals with NP. Effects of the intraperitoneal administration of VEH, HRW at 0.018 μmol, JWH-133 at 2 mg/kg, or the combination of both treatments on the mechanical allodynia (**A**), thermal hyperalgesia (**B**), and cold allodynia (**C**) provoked by CCI in the ipsilateral paw are shown. The effects of treatments are represented in the *y*-axis as maximal possible effect (%) for (**A**) mechanical allodynia and (**B**) thermal hyperalgesia and as inhibition (%) for (**C**) cold allodynia. In each test, symbols denote significant differences (*p* < 0.05) versus * VEH-VEH, + HRW-VEH, $ VEH-JWH-133. A one-way ANOVA followed by a Student-Newman-Keuls post hoc test was performed. The data are represented as mean ± SEM; n = 6 animals for each experimental group.

The antiallodynic and antihyperalgesic activities produced by the intraperitoneal administration of low doses of HRW (0.018 μmol) and JWH-133 (2 mg/kg), given alone and combined, were evaluated (Figure 4). The combined treatment of HRW and JWH-133 showed a significantly greater inhibition of the mechanical allodynia (*p* < 0.0001, one-way ANOVA; Figure 4A), thermal hyperalgesia (*p* < 0.0001, one-way ANOVA; Figure 4B), and cold allodynia (*p* < 0.0001, one-way ANOVA; Figure 4C) compared to the effects produced by either treatment administered alone. In addition, the antihyperalgesic actions produced by HRW alone were higher than those produced by VEH, and those of JWH-133 were higher than those of VEH and HRW given separately (Figure 4B), and the cold antiallodynic actions of HRW and JWH-133 were also higher than those produced by VEH (Figure 4C).

Specifically, the combined treatment achieved an 83% inhibition of the mechanical allodynia, in contrast with the 10% and 32% inhibition of HRW and JWH-133 given alone, respectively (Figure 4A). Similar effects were observed for thermal hyperalgesia with values of 65%, 34%, and 45% of inhibition and for cold allodynia with values of 74%, 38%, and 35% inhibition induced by HRW and JWH-133 combined and by HRW and JWH-133 administered individually, respectively (Figure 4B,C). These results revealed a positive interaction between HRW and JWH-133 in inhibiting NP.

### 2.6. The Antinociceptive Effects of the Co-Treatment of HRW and Pregabalin in Animals with NP

**Figure 5:**
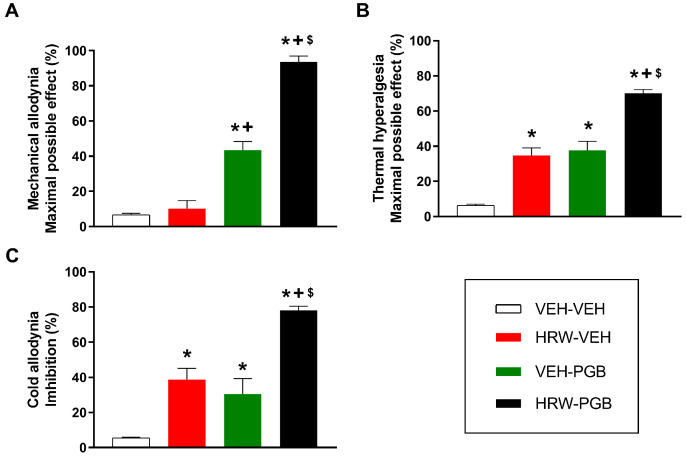
The analgesic actions of the combined treatment of HRW and pregabalin (PGB) in animals with NP. Effects of the intraperitoneal administration of VEH or HRW at 0.018 μmol, pregabalin (PGB) at 10 mg/kg, or the combination of both treatments on the mechanical allodynia (**A**), thermal hyperalgesia (**B**), and cold allodynia (**C**) provoked by CCI in the ipsilateral paw are shown. The effects of treatments are represented in the *y*-axis as the maximal possible effect (%) for (**A**) mechanical allodynia and (**B**) thermal hyperalgesia and as inhibition (%) for (**C**) cold allodynia. In each test, each symbol denotes significant differences (*p* < 0.05) versus * VEH-VEH, + HRW-VEH, $ VEH-PGB. A one-way ANOVA followed by a Student-Newman-Keuls post hoc test was performed. The data are represented as mean ± SEM; n = 6 animals for each experimental group.

We further investigated whether HRW could potentiate the effects of pregabalin, a first-line treatment for NP. CCI mice received either HRW (0.018 μmol), pregabalin (10 mg/kg), or their combination. The results indicated that HRW significantly enhanced the antinociceptive properties of pregabalin, with greater inhibition of the mechanical allodynia (*p* < 0.0001, one-way ANOVA; Figure 5A), thermal hyperalgesia (*p* < 0.0001, one-way ANOVA; Figure 5B), and cold allodynia (*p* < 0.0001, one-way ANOVA; Figure 5C) when compared to the effects produced by each treatment given alone.

The combined treatment achieved 93% inhibition of mechanical allodynia, in contrast with 10% for HRW or 43% for PGB alone (Figure 5A). For thermal hyperalgesia values of 70%, 34%, and 37% (Figure 5B) and for cold allodynia values of 78%, 38%, and 29% of inhibition induced by HRW and pregabalin combined and by HRW and pregabalin given alone were obtained (Figure 5C). These findings revealed a positive interaction between HRW and pregabalin in inhibiting the allodynia and hyperalgesia provoked by CCI.

These data also confirmed that the mechanical antiallodynic effects produced by this dose of pregabalin are higher than those of VEH or HRW alone and that the antihyperalgesic and cold antiallodynic actions of HRW and pregabalin administered separately were greater than those produced by VEH.

In all tests, HRW, JWH-133, or pregabalin administered separately or combined did not produce any effect in the ipsilateral paws of SHAM-operated mice.

### 2.7. Expression of 4-HNE, NLRP3, p-ERK 1/2, and p-AKT in the DRG of CCI Mice

**Figure 6:**
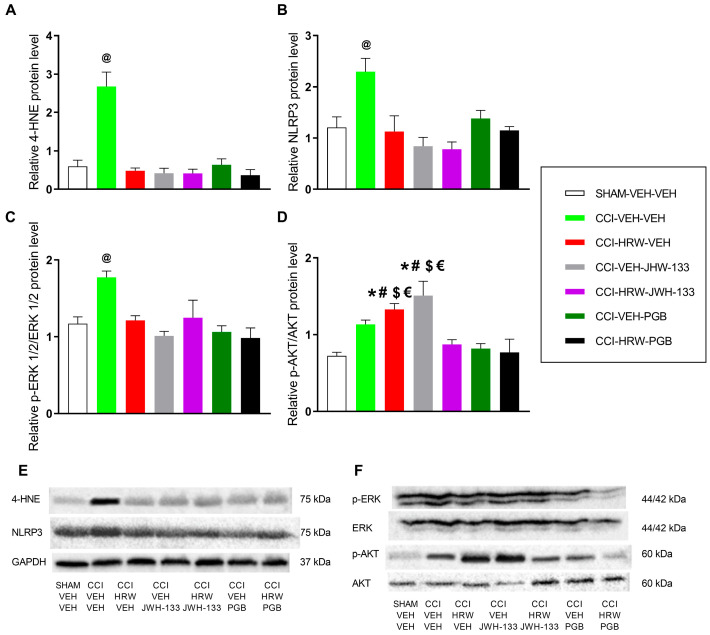
Effects of treatment with HRW, JWH-133, or pregabalin alone and combined on the expression of 4-HNE, NLRP3, p-ERK 1/2, and p-AKT in the DRG of CCI mice. Results are shown on the *y*-axis as relative levels of (**A**) 4-HNE, (**B**) NLRP3, (**C**) p-ERK 1/2, and (**D**) p-AKT in the DRG of CCI mice treated with 0.08 μmol HRW (CCI-HRW-VEH), 2 mg/kg JWH-133 (CCI-VEH-JWH-133), the combination of HRW with JWH-133 (CCI-HRW-JWH-133), 10 mg/kg pregabalin (PGB) (CCI-VEH-PGB), or the combination of HRW with pregabalin (CCI-HRW-PGB). The expression of these proteins in CCI-VEH-VEH and SHAM-VEH-VEH is also shown. Protein levels are normalized to GAPDH for 4-HNE and NLRP3 and to total ERK 1/2 and AKT for p-ERK 1/2 and p-AKT, respectively. Representative blots (**E**) for 4-HNE, NLRP3, and GAPDH and (**F**) for p-ERK 1/2, ERK 1/2, p-AKT, and AKT are shown. In each graph, symbols denote significant differences: @ vs. all the other groups, * vs. SHAM-VEH-VEH, # vs. CCI-HRW-JWH-133, $ vs. CCI-VEH-PGB, and € vs. CCI-HRW-PGB. A one-way ANOVA analysis followed by a Student-Newman-Keuls post hoc test was performed. A sample size of n = 3 was used for each experimental group.

We evaluated the expression of 4-HNE, an indicator of oxidative stress; NLRP3, an inflammatory marker; p-ERK, an indicator of neuronal excitability; and p-AKT, a marker of nociceptive signaling in the DRG of CCI mice treated with HRW, JWH-133, or pregabalin alone and combined with HRW. Results suggested that CCI increased the expression of 4-HNE (*p* < 0.0001, one-way ANOVA; Figure 6A), NLRP3 (*p* < 0.0004, one-way ANOVA; Figure 6B), and p-ERK 1/2 (*p* < 0.0004, one-way ANOVA; Figure 6C) compared to SHAM-operated mice. Moreover, the administration of HRW, JWH-133, or pregabalin, either alone or in combination, significantly reduced the overexpression of 4-HNE (Figure 6A), NLRP3 (Figure 6B), and p-ERK 1/2 (Figure 6C) observed in the DRG of CCI mice treated with VEH (CCI-VEH-VEH).

Regarding p-AKT, CCI mice showed a tendency towards increased levels compared to SHAM-VEH-VEH mice, although this difference was not statistically significant (Figure 6D). Nevertheless, treatment with HRW or JWH-133 alone increased the expression of p-AKT as compared to those detected in SHAM animals treated with VEH and in CCI mice treated with HRW plus JWH-133, pregabalin alone, and combined with HRW (*p* < 0.0001, one-way ANOVA; Figure 6D), but not with CCI mice treated with VEH.

## 3. Discussion

NP is a highly prevalent and disabling condition that affects millions of people worldwide. However, the prolonged use of the most commonly prescribed treatments is often associated with several side effects, which can lead patients to discontinue medication. In this study, we investigate new combined treatments with HRW for the management of NP, aiming to reduce the high doses or the long-time administration of established treatments, such as pregabalin, or of tentative therapies, such as CB2R agonists.

In a CCI mouse model, our results indicated that HRW significantly inhibited the mechanical allodynia, thermal hyperalgesia and cold allodynia in a dose-dependent manner. This is particularly relevant as, to our knowledge, this is the first time that a dose–response curve for HRW has been established in a CCI-induced NP model. Regarding JWH-133, a CB2R agonist, or pregabalin, an anticonvulsant drug, both drugs also exhibited dose-dependent antinociceptive effects in CCI mice. These results are in line with previous studies reporting that CB2R agonists, such as JWH-015, dose-dependently reduced the hypersensitivity induced by peripheral nerve injury in rodents [23]. Similarly, pregabalin had been reported to exert dose-dependent antiallodynic effects following its intraperitoneal administration in a mouse model of spared nerve injury, with similar ranges of doses tested by us (3–30 mg/kg) [24]. Our findings further revealed the dose–response inhibitory effects induced by both drugs in other nociceptive responses, such as thermal hyperalgesia and cold allodynia provoked by CCI.

Our study further demonstrated that the co-administration of a low dose of HRW potentiated the analgesic effects produced by JWH-133 and pregabalin. That is, the combination of HRW and JWH-133 significantly enhanced the mechanical and thermal antiallodynic and thermal antihyperalgesic effects produced by each treatment given alone. This improvement is higher in the inhibition of allodynia than hyperalgesia. That is, the mechanical and thermal antiallodynic effects produced by HRW combined with JWH-133 are around 83% and 74%, respectively, in contrast to the 65% antihyperalgesic effects produced by this combined treatment. Suggesting that this combined treatment is more effective in improving the antiallodynic than the antihyperalgesic properties of JWH-133 under NP conditions. Other mixture treatments involving JWH-133 have also been assessed; for example, its co-administration with hydrogen sulfide (H_2_S) donors also improved the pain reliever effects produced by this CB2R agonist in animals with NP [25]. Furthermore, a synergistic interaction between JWH-015, another CB2R agonist, and morphine, a µ opioid receptor agonist, inhibiting NP induced by the spared nerve injury of the sciatic nerve has also been reported [26]. Nevertheless, this study provides, for the first time, evidence of a positive interaction between H_2_ and the CB2R system in the modulation of sciatic nerve injury–induced NP in mice. Given the favorable safety profile of H_2_ compared with the well-known adverse effects of morphine, its co-administration with JWH-133 may represent a safer and more effective therapeutic strategy for the management of NP.

Our findings also demonstrated that a low dose of HRW can enhance the analgesic effectiveness of pregabalin, a first-line treatment for NP. The mechanical and thermal antiallodynic and antihyperalgesic actions produced by this drug combination were greater than those made by either treatment administered individually. Other combinations involving pregabalin have also been explored. For example, the co-administration of pregabalin with antidepressants, for instance tricyclics or selective serotonin reuptake inhibitors, has been reported to effectively alleviate diabetic peripheral NP. Similarly, co-treatment of pregabalin with opioids has also shown analgesic efficacy, although addiction issues remain a concern [27]. In preclinical models of NP, the co-administration of cobalt protoporphyrin IX (CoPP), an HO-1 inducer, and/or carbon monoxide-releasing molecule 2 (CORM-2) has been shown to potentiate the antiallodynic effects of pregabalin in spared nerve-injury induced NP in mice [24]. Our results reported, for the first time, that pregabalin in combination with HRW results in a greater analgesic action, especially inhibiting mechanical allodynia, where the combined treatment achieved 93% inhibition versus those of 70% and 78% achieved in reducing thermal hyperalgesia and cold allodynia, respectively. The present findings highlight the combination of HRW with pregabalin as particularly advantageous compared with other pregabalin-based strategies previously reported. Although agents such as CoPP and CORM-2 have been reported to enhance pregabalin’s efficacy, their potential toxicity and limited translational applicability raise concerns regarding long-term safety. In contrast, HRW offers a well-tolerated source of H_2_ with established antioxidant and anti-inflammatory properties, and it has been shown to exert these effects without notable side effects [22].

It is well established that oxidative stress is a critical contributor to the pathogenesis of NP. In the present study, we observed a marked increase in the expression of 4-HNE within the DRG of CCI mice. This alteration was normalized following treatment with HRW, JWH-133, or pregabalin administered individually. Evidence from previous studies supports the antioxidant properties of these agents when administered individually. Specifically, H_2_ has been reported to exert part of its analgesic effects by neutralizing cytotoxic ROS [19,28] and by normalizing the overexpression of the oxidative stress marker 4-HNE while upregulating antioxidant enzymes such as SOD-1 in DRG of mice with NP [29].

Similarly to H_2_, activation of CB2R also attenuates oxidative stress by reducing ROS production, lipid peroxidation, and 4-HNE expression in the spinal cord of mice with oxaliplatin-induced NP [30] and in in vitro models of osteoarthritis [31]. Consistently, CB2R agonists have been shown to activate the Nrf2-mediated endogenous antioxidant pathway; for example, macrophages derived from mice with diet-induced obesity exhibit increased Nrf2 activity following CB2R stimulation [32]. Pregabalin also exerts part of its palliative effects by modulating the expression of antioxidant enzymes, including HO-1 and Arg-1 [24]. Our data further showed that the combined administration of HRW with either JWH-133 or pregabalin fully restored the elevated 4-HNE levels to baseline, suggesting that all treatments converge on a common redox-related mechanism. The simultaneous normalization of 4-HNE by all agents may reflect a broader and more efficient rebalancing of redox homeostasis, which may underlie the enhanced analgesic efficacy of the combined treatments. Nevertheless, other complementary mechanisms might be involved in the superior analgesic effects observed with the combined treatments, likely related to inflammation, plasticity changes, or nociceptive pathways activated during NP.

H_2_ targets core pathways such as NLRP3 inflammasome and MAPK to regulate and suppress inflammation [22]. Accordingly, the increased expression of NLRP3 induced by CCI in the DRG was inhibited by HRW. Since NLRP3 activation is promoted by oxidative stress, the reduction in ROS by HRW may also contribute to the attenuation of inflammatory responses [28,33]. In parallel, CB2R agonists have been shown to promote NLRP3 degradation through autophagy in spinal cord injury models [6] and to exert peripheral anti-inflammatory effects by limiting immune cell migration and pro-inflammatory cytokine release at the injury site [32,34]. Similarly, pregabalin reduces NLRP3 expression, and its analgesic actions are associated with suppressed microglial activation and reduced levels of pro-inflammatory cytokines, including TNF-α [24]. Consistently, our results indicated that administration of JWH-133 or pregabalin, either alone or in combination with HRW, suppressed the overexpression of this inflammasome in the DRG of animals with NP. Therefore, the superior analgesic outcomes observed suggest that the anti-inflammatory effects of HRW, JWH-133, and pregabalin given alone reinforced the analgesic properties of the combined therapies.

Hypersensitivity following nerve injury in different NP models correlates with increased phosphorylation of ERK, which is associated with synaptic plasticity in neurons, leading to abnormal signaling and increased excitability [35]. Molecular analysis revealed that the expression of p-ERK increased in the DRG of animals with NP, thus contributing to the hypersensitivity manifested at 28 days after injury. Moreover, all individual treatments and combinations normalized the elevated p-ERK levels in the DRG of CCI mice. In agreement, HRW inhibits ERK 1/2 activation in the DRG of animals with chemotherapy-induced NP [36], and the reduced expression of p-ERK 1/2 induced by CB2R activation contributed to the antinociceptive actions in a rat model of NP [37]. In the same way, the antinociceptive mechanisms of pregabalin have also been associated with the inhibition of MAPK activation, such as reduced p-ERK 1/2 levels in spinal neurons, as demonstrated [38,39]. This study revealed that the co-administration of HRW with either JWH-133 or pregabalin also attenuated the nerve injury–induced up-regulation of p-ERK1/2 in the DRG of CCI mice, a signaling node critically involved in central and peripheral sensitization. This downregulation is consistent with the robust antihyperalgesic effects observed with both combined treatments. However, similar to what was observed for 4-HNE and NLRP3, no additional reduction in p-ERK1/2 expression was detected with the combined therapy compared with each compound administered individually. A likely explanation is that both HRW, JWH-133, and pregabalin fully normalized p-ERK1/2 levels to baseline when administered alone, thereby reaching a physiological floor beyond which further suppression is not achievable.

AKT phosphorylation, a regulator of nociceptive processing, plays an important role in the transmission of pain signals within the spinal cord and peripheral sensory pathways [10,40]. In our study, however, p-AKT regulation presented a complex pattern. Although CCI alone did not markedly elevate p-AKT, both HRW and JWH-133 administered as monotherapies increased its expression above SHAM levels. This response contrasts with the inhibitory effect of HRW on p-AKT reported in a paclitaxel-induced neuropathy model [36], yet is consistent with evidence that CB2R activation can enhance AKT phosphorylation in immune cells [34,41]. Such discrepancies may reflect NP model-specific differences, and it is also possible that the relatively low dose of HRW used here was insufficient to counteract subtle CCI-induced changes in AKT signaling within the DRG. Notably, when HRW and JWH-133 were co-administered, p-AKT levels returned to baseline despite the increases produced by each agent alone. This normalization suggests that the combined treatment may rebalance upstream regulatory inputs such as oxidative stress, inflammatory mediators, or CB2-related signaling that converge on the AKT pathway, thereby preventing compensatory activation and reducing AKT-dependent nociceptor sensitization. Although this theory should be interpreted cautiously, this coordinated suppression of AKT phosphorylation aligns with the superior analgesic effects observed with the combination therapy.

Our data further showed that pregabalin, whether administered alone or in combination with HRW, fully normalized p-AKT levels in the DRG of CCI mice. This reduction in AKT phosphorylation indicates an effective suppression of nerve-injury–induced neuronal sensitization and downstream nociceptive signaling, which contributes to the greater antinociceptive response observed with the combined treatment. Overall, these findings suggest that concomitant modulation of redox homeostasis, neuroinflammation, and the p-ERK1/2 and p-AKT signaling cascades induced by these combined therapies may recruit a broader, more integrated regulatory network that ultimately enhances its analgesic efficacy during NP.

The greater analgesic efficacy observed with the combination of HRW and JWH-133 or pregabalin highlights several promising avenues for clinical application. By simultaneously attenuating oxidative stress, inflammatory signaling, and maladaptive nociceptive pathways, HRW could enable effective pain control with lower doses of conventional analgesics, an approach that could significantly reduce the burden of adverse effects associated with chronic high-dose treatments in patients with NP.

However, some limitations of this study, such as the use only in male mice without addressing potential sex differences in pain mechanisms, oxidative stress, or drug responses, and the fact that it only evaluated the effects of acute intraperitoneal administration of these co-treatments without assessing the potential long-term adverse effects, oral bioavailability, and dosing equivalence, limit its translational and therapeutic relevance.

## 4. Materials and Methods

### 4.1. Animal Experimental Models

All animal care and experimental procedures were approved by the local Committee of Animal Use and Care of the Autonomous University of Barcelona (ethical code: 4581) and conducted in accordance with the guidelines of the European Commission’s directive (2010/63/EC) and the Spanish Law (RD 53/2013) regulating animal research.

Male C57BL/6 mice (5–6 weeks old) were obtained from Envigo Laboratories (Barcelona, Spain). Animals were group-housed under controlled conditions, at a 22 °C temperature and 66% humidity environment, exposed to a 12-h light/dark cycle and unlimited access to water and food. Four male mice were kept in polypropylene cages outfitted with a carton hut and cellulose fragments to create an enriching atmosphere.

Behavioral testing was performed from 9:00 a.m. to 5:00 p.m. following a one-week acclimatization period to the housing conditions. Animals were also acclimated to the testing room for 1 h before the commencement of the tests. For each test, the order of animals being tested was randomized daily, and each subject was tested at a different time on each test day. For each animal, two distinct investigators were involved: the primary investigator (SENF) administered the treatment in accordance with the randomization table. This researcher was uniquely cognizant of the treatment group allocation. An additional investigator (NAT), blinded to the treatment, evaluated the mechanical allodynia, thermal hyperalgesia, and cold allodynia of these subjects.

All efforts were made to minimize the animal suffering and to reduce the number of animals used in accordance with the 3Rs principles (replacement, reduction, and refinement). The number of animals used in each group was 6 (n = 6). Animals were randomly assigned to each experimental group. Random numbers were generated using the standard = RAND ( ) function in Microsoft Excel. A total of 372 mice were used in this study.

### 4.2. Neuropathic Pain Model

To induce NP, a CCI of the sciatic nerve was performed in mice. Surgical procedures were carried out under isoflurane anesthesia (3% for induction and 2.5% for maintenance). After confirming the loss of the hind limb withdrawal reflex, the surgical site was shaved. Afterward, the biceps femoris and the gluteus superficialis were dissected, thereby exposing the sciatic nerve. Three ligatures were placed around the nerve using 4/0 silk sutures (Laboratorio Aragó, SL, Barcelona, Spain), spaced approximately 1 mm apart, ensuring the epineural circulation was not obstructed. SHAM-operated control mice underwent the same surgical procedure, excluding nerve ligation.

### 4.3. Nociceptive Tests

Mechanical allodynia was tested by evaluating the hind limb withdrawal reflex in response to von Frey filaments of increasing stiffness (0.4 to 3 g, North Coast Medical, Inc., San Jose, CA, USA). Mice were individually placed in transparent Plexiglas cylinders (20 cm height × 9 cm diameter) on an elevated wire mesh platform, allowing access to the plantar area of the hind paw.

The up-down method [42] was used, starting with the 0.4 g filament. If a withdrawal response was observed, the filament with lower force was applied next; otherwise, a higher force filament was used. The threshold of the response was calculated using an Excel program (Microsoft Iberia, SRL, Barcelona, Spain) incorporating curve fitting of the data.

Thermal hyperalgesia was assessed using the plantar test (Ugo Basile, Varese, Italy), which applies a radiant heat stimulus to the hind paw to measure paw withdrawal latency. Mice were individually placed in transparent Plexiglas cylinders (20 cm height × 9 cm diameter) on a glass platform. A heat source was applied under the plantar surface of the hind paw until a withdrawal response was observed or until 12 s (cut-off) were reached to prevent tissue damage. Three measurements were taken for each animal, and the mean paw withdrawal latency was calculated.

Cold allodynia was tested using the cold plate test (Ugo Basile, Varese, Italy). A constant cold stimulus at 5 °C was applied for 300 s, during which the number of hind paw lifts was recorded.

Both the contralateral and ipsilateral hind paws were assessed in all nociceptive tests.

### 4.4. Pharmacological Treatments

HRW was freshly generated using an HRW generator (Hydrogen, Osmo-star Soriano S.L., Alicante, Spain) at a concentration of 0.3 mM (300 µmol/L), equivalent to 0.3 µmol/mL. Accordingly, the administered doses of 0.018, 0.036, 0.075, and 0.150 µmol for a 25 g mouse, represent approximately 0.72, 1.44, 3.0, and 6.0 µmol/kg.

Given the high volatility of H_2_, HRW was generated individually for each animal, and immediately injected to minimize gas dissipation. In addition, the concentration of dissolved H_2_ was verified immediately before injection using an electrode, ensuring that the intended H_2_ concentration was maintained at the moment of administration.

JWH-133 (Sigma-Aldrich, St. Louis, MO, USA) was dissolved in a saline solution with 1% Tween 80 (Sigma-Aldrich, St. Louis, MO, USA), and pregabalin (Sigma-Aldrich, St. Louis, MO, USA) was dissolved in a saline solution (0.9% NaCl).

For the dose–response curve experiments, HRW was administered at doses of 0.018, 0.036, 0.075, and 0.150 μmol. JWH-133 at 1, 2, 3, 5, 10, and 20 mg/kg obtained from a stock solution of 100 mg/kg; and pregabalin at 5, 10, 20, and 30 mg/kg obtained from a stock solution of 50 mg/kg.

Based on the dose–response curve results, 0.018 μmol of HRW was combined with 2 mg/kg of JWH-133 or 10 mg/kg of pregabalin. These doses were selected as the lowest of each drug that produced significant inhibitory effects across all tests.

The sample size calculation was performed using G*Power 3.1.7 software. Based on pilot data and assuming an α risk of 0.05 and a β risk of 0.2 in a two-tailed test, six animals per group were calculated as sufficient to detect statistically significant differences. Every effort was made to minimize animal suffering and reduce the number of animals used; therefore, tissues from animals subjected to behavioral testing were also utilized for Western blot analyses.

All drugs were prepared right before their use, and for each group treated with a drug, the corresponding control group received the same volume of the respective vehicle (VEH). In accordance with previous studies [21,29], HRW was intraperitoneally administered at 1 h before the tests. JWH-133 and pregabalin, given alone and combined with HRW, were also intraperitoneally injected in a final volume of 10 mL/kg and at 1 h prior to testing, based on previous findings [25].

### 4.5. Experimental Design

In this study, a mouse model of CCI of the sciatic nerve was used. Mechanical allodynia, thermal hyperalgesia and cold allodynia were tested in that order at the following time points: before surgery (baseline) and at day 28 post-surgery (CCI/SHAMP) for evaluation of the effects of treatments alone and combined. After tests, animals were euthanized by cervical dislocation, and the DRG samples from SHAM and CCI mice treated with VEH, HRW, JWH-133, pregabalin, HRW plus JWH-133, and HRW plus pregabalin were obtained. Western blot analysis was performed to evaluate the possible modulation of oxidative stress, inflammatory reactions, plasticity changes, and nociceptive pathways induced by these treatments.

### 4.6. Western Blotting

Twenty-eight days post-surgery, SHAM and CCI mice were euthanized by cervical dislocation, and DRG were collected and stored at −80 °C until use. DRG samples from two animals of the same group were pooled to obtain sufficient material for protein extraction and robust immunoblotting. Each pooled sample was subsequently lysed in RIPA buffer (Sigma-Aldrich, St. Louis, MO, USA), containing 1% phosphatase and 0.5% protease inhibitors, followed by sonication for 10 s. Samples were then incubated for 1 h at 4 °C to allow solubilization, and then a second sonication was performed. Afterward, samples were centrifuged for 20 min at 4 °C and 700× *g*, and the supernatants were collected. Protein concentration of the samples was determined using the Bradford method with the Bio-Rad Protein Assay Dye Reagent Concentrate (Bio-Rad Laboratories, Hercules, CA, USA), following the manufacturer’s instructions.

Protein samples (60 μg) were mixed with 4× Laemmli loading buffer, denatured by heating at 95 °C, and then separated on 4% stacking/12% separating sodium dodecyl sulfate-polyacrylamide gels using Tris-Glycine SDS-PAGE by electrophoresis. Proteins were then transferred to PVDF membranes for 2 h, which were blocked for 75 min with blocking buffer and then incubated overnight in agitation at 4 °C with the primary antibodies listed in Table 2. GAPDH was used as a loading control. Subsequently, membranes were incubated for 1 h at room temperature with horseradish peroxidase (HRP)-conjugated secondary antibodies: anti-rabbit (1:5000) for AKT, ERK ½, GAPDH, 4-HNE, p-AKT, and p-ERK 1/2, and anti-mouse (1:5000) for NLRP3. A buffer solution consisting of either Tris-buffered saline (TBS) with Tween 20 and non-fat dry milk (5%) or bovine serum albumin (BSA) (5%), or phosphate-buffered saline (PBS) with Tween 20 and non-fat dry milk (5%) solution was used for membrane blocking, primary antibody and secondary antibody dilutions in the incubation steps. Finally, the protein bands were detected using chemiluminescence (ECL kit; GE Healthcare, Little Chalfont, Buckinghamshire, UK) and quantified by densitometry using the ImageJ software (version 1.8.0; National Institutes of Health, Bethesda, MD, USA).

### 4.7. Statistical Analysis

Statistical analysis and graphs were generated using the GraphPad Prism 8.0 software (La Jolla, CA, USA) and the Statistical Package for Social Sciences (SPSS; version 28, IBM, Madrid, Spain). The obtained values are presented in the graphs as mean ± standard error of mean values (SEM). The statistical approach assumptions were evaluated using Bartlett’s test for variance homogeneity and the Shapiro–Wilk test for data distribution.

Data were analyzed using a Student’s *t*-test when comparing two groups, such as in the validation experiments of the CCI model. Data were evaluated using a one-way analysis of variance (ANOVA) when multiple groups were involved, such as in the dose–response analysis and combined treatment experiments. When the ANOVA resulted in significance, we conducted the Student-Newman-Keuls post hoc test to identify differences between doses or groups. For all tests, results with a *p* < 0.05 were considered statistically significant.

In the von Frey filaments and plantar tests, antinociception is expressed as the percentage of maximal possible effect, where the test latencies pre-drug (baseline) and post-drug administration are compared and calculated in accordance with the following equation:

Maximal possible effect (%) = ((drug − baseline)/(cut-off − baseline)) × 100

In the cold plate test, antinociception is expressed according to the following equation:

Inhibition (%) = ((number of paw elevations at baseline − number of paw elevations after drug)/(number of paw elevations at baseline)) × 100.

Cutoffs of 3 g and 12 s for the mechanical allodynia and thermal hyperalgesia were employed.

## 5. Conclusions

The results of this study show that HRW improves the analgesic effects of either pregabalin or JWH-133 when taken together. These positive effects are mediated, at least in part, by the attenuation of nociceptive signaling and neuronal hyperexcitability, as well as the reduction in inflammation and oxidative stress. Despite the need for more research studies, these results suggest that HRW is a promising supplement to gabapentinoid and cannabinoid treatments, with the potential to increase efficacy and decrease side effects associated with high doses of these drugs, ultimately improving patient quality of life.

## Figures and Tables

**Table 1:** Validation of the CCI model. Evaluation of mechanical allodynia (g), thermal hyperalgesia (s), and cold allodynia (number) in CCI-VEH and SHAM-VEH mice at 28 days post-surgery.

Group	Mechanical Allodynia (Von Frey Filament Strength, g)	Thermal Hyperalgesia (Withdrawal Latency, s)	Cold Allodynia (Paw Lifts, Number)
SHAM-VEH	2.67 ± 0.06	11.89 ± 0.20	0.33 ± 0.52
CCI-VEH	0.38 ± 0.18 *	3.62 ± 0.39 *	9.83 ± 2.71 *

The * symbol denotes significant differences between CCI-VEH and SHAM-VEH. An unpaired Student’s *t*-test analysis was performed for each comparison. A *p* < 0.05 was considered statistically significant. The obtained values are presented as mean ± SEM; n = 6 animals were used for each experimental group.

**Table 2:** Primary antibodies used for Western blot analysis and their dilutions.

Primary Antibody	Dilution	Commercial Supply
4-HNE	1:100	Abcam, Cambridge, UK
NLRP3	1:200	Adipogen Life Sciences, Epalinges, Switzerland
p-ERK	1:250	Cell Signaling Technology, Danvers, MA, USA
ERK 1/2	1:250	Cell Signaling Technology, Danvers, MA, USA
p-AKT	1:200	Cell Signaling Technology, Danvers, MA, USA
AKT	1:200	Cell Signaling Technology, Danvers, MA, USA
GAPDH	1:5000	Merck, Billerica, MA, USA

## Data Availability

Data is contained within the article.
